# Photosensitized Oxidative Damage from a New Perspective: The Influence of Before-Light and After-Light Reaction Conditions

**DOI:** 10.1021/acs.joc.4c01305

**Published:** 2024-09-04

**Authors:** Lloyd Lapoot, Shakeela Jabeen, Ryan M. O’Connor, Witold Korytowski, Albert Girotti, Alexander Greer

**Affiliations:** † Department of Chemistry, Brooklyn College of the City University of New York, Brooklyn, New York 11210, United States; ‡ Ph.D. Program in Biochemistry, The Graduate Center of the City University of New York, 365 Fifth Avenue, New York, New York 10016, United States; § Ph.D. Program in Chemistry, The Graduate Center of the City University of New York, 365 Fifth Avenue, New York, New York 10016, United States; ∥ Department of Biophysics, Jagiellonian University, Gołębia 24 Street, 31-007 Kraków, Poland; ⊥ Department of Biochemistry, Medical College of Wisconsin, Milwaukee, Wisconsin 53226, United States

## Abstract

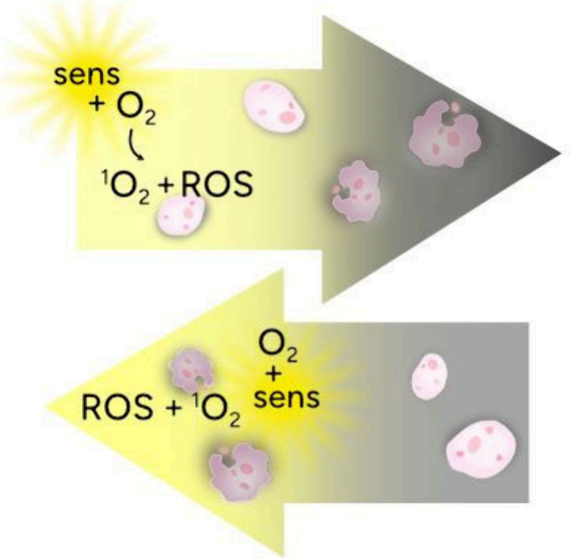

Photooxidative damage is heavily influenced by the presence of bioactive agents. Conversely, bioactive agents influence the local environment, which in turn is perturbed by photooxidative damage. These sorts of processes give rise to a version of the “chicken-and-egg” quandary. In this Perspective, we probe this issue by referring to photooxidative damage in one direction as the light-dark (L-D) sequence and in a second direction as the dark-light (D-L) sequence with a reversed cause and effect. The L-D sequence can lead to the downstream production of reactive molecular species (RMS) in the dark, whereas the D-L sequence can be a pre-irradiation period, such as an additive to limit cellular iron levels to enhance biosynthesized amounts of a protoporphyrin sensitizer. A third direction comes from L-D or D-L sequences, or both simultaneously, which can also be useful for optimizing photodynamics. Photodynamic optimization will benefit from understanding and quantitating unidirectional L-D and D-L pathways, and bidirectional L-D/D-L pathways, for improved control over photooxidative damage. Photooxidative damage, which occurs during anticancer photodynamic therapy (PDT), will be shown to involve RMS. Such RMS include persulfoxides (R_2_S^+^OO^–^), NO_2_^•^, peroxynitrate (O_2_NOO^–^), OOSCN^–^, SO_3_^•–^, selenocyanogen [(SeCN)_2_], the triselenocyanate anion [(SeCN)_3_^–^], I^•^, I_2_^•–^, I_3_^–^, and HOOI, as well as additives to destabilize membranes (e.g., caspofungin and saponin A16), inhibit DNA synthesis (5-fluorouracil), or sequester iron (desferrioxamine). In view of the success that additive natural products and repurposed drugs have had in PDT, a Perspective of additive types is expected to reveal mechanistic details for enhanced photooxidation reactions in general. Indeed, strategies for how to potentiate photooxidations with additives remain highly underexplored.

## Introduction

This Perspective describes work from the authors’ laboratories and literature on the proliferation of photooxidative reactions in the presence of adjuvants. Reactive oxygen species (ROS) are formed in photosensitized oxidation reactions; however, pathways to optimize photodynamics are needed for improved damage control. A synopsis of this subject could unveil factors that control negative side effects of photosensitized oxidation reactions.

**Figure 1:**
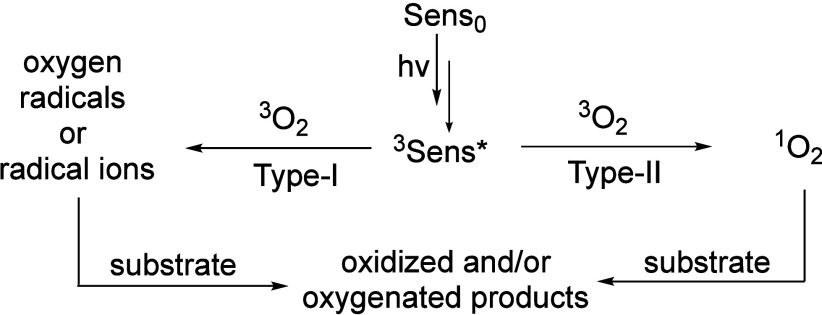
Upon exposure to light, the sensitizer is excited, which in the presence of oxygen forms oxygen radicals and radical ions by Type-I processes or singlet oxygen (^1^O_2_) by a Type-II process leading to oxidized and oxygenated products.

Photosensitized oxidation is defined as shown in Figure 1.^1−5^ In the first case, free radical species arise from Type-I electron and/or H atom transfer from triplet photoexcited sensitizer to ground state oxygen (e.g., HO^•^, O_2_^•**–**^, HO_2_^•^, and H_2_O_2_). In the second case, Type-II energy transfer leads to the formation of the nonradical, singlet molecular oxygen (^1^O_2_). Unsaturated lipid additives such as cholesterol can react by both Type-I and Type-II processes.^6−11^ As will be discussed later, additives in photooxidation reactions can lead to reactive molecular species (RMS, where M are species containing not only oxygen, but also nitrogen, sulfur, selenium, and iodine). RMS can range from nitrite radical and sulfite radical anion to iodide radical and iodine radical anion. We will also see that additives can induce phototoxicity by membrane disruption, DNA intercalation, enzyme inhibition, and/or sequestration.

**Figure 2:**
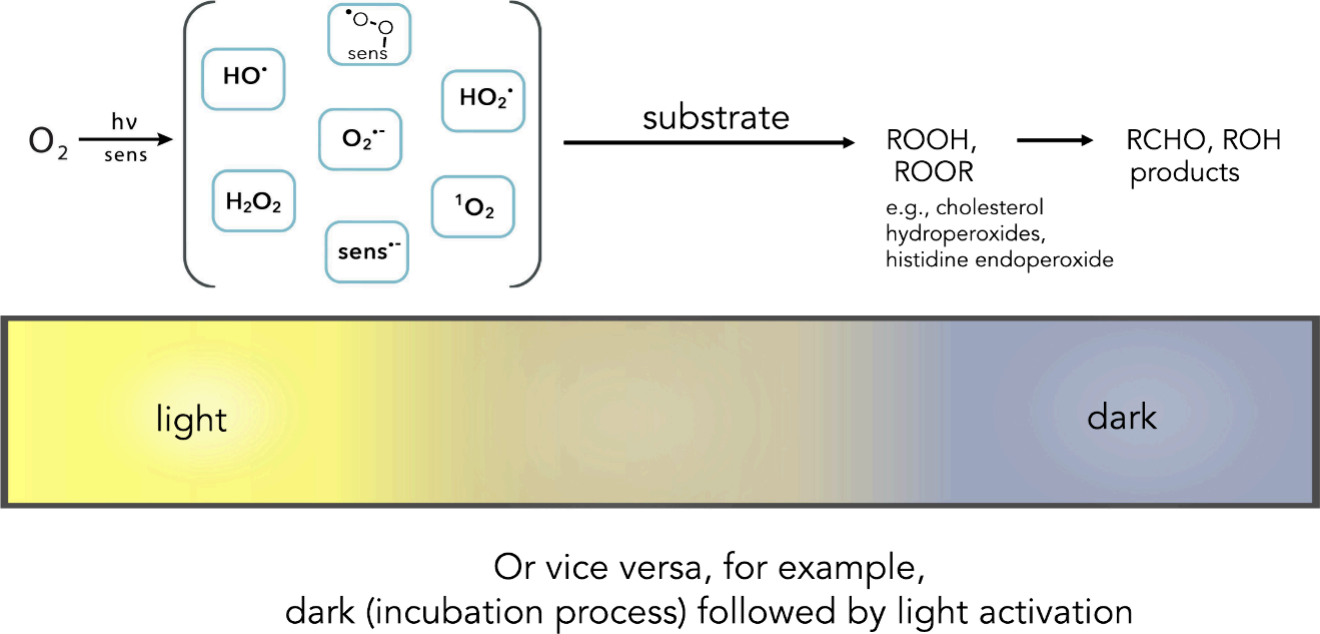
Light-dark (L-D) reaction sequence with ROS from Type-I or Type-II photosensitized oxidation followed by RMS production for overall amplification of reactivity. In the L-D sequence, various RMS initially form; an example of sens-OO^•^ is the peroxyl radical formed in the self-sensitized photooxidation of toluidine blue O. An opposite reaction sequence can occur, namely a dark-light (D-L) reaction sequence. Middle ground where L-D and/or D-L sequences may arise, such as the integrity of the cell membrane being compromised for greater cell permeability, where in turn sensitizer uptake is higher for greater photooxidative damage.

Assessing additives’ influence is based on consideration of photooxidative damage in one direction [light-dark (L-D) sequence] and in a second direction [dark-light (D-L) sequence] (Figure 2). In the first case, the light event leads to the production of RMS, then subsequent reactions take place thermally (in the dark). In the second case, a dark event is a post-irradiation period, but also can be a pre-irradiation period such as compound incubation prior to the photoproduction of RMS. An example of the D-L sequence is an additive in the dark limiting the cellular iron level to enhance the amount of protoporphyrin (PpIX) sensitizer biosynthesized for an increased photochemical step. Another example of the D-L sequence is to inhibit the export of PpIX. This is also relevant for pre-existing sensitizers, where inhibiting their export will lead to an increased photochemical step in the D-L sequence.

The “chicken and egg” problem of photooxidative damage and the influence of additives or adjuvants in this regard is the subject of this Perspective. The Perspective attempts to uncover how compounds can elicit increased photooxidative damage upon their addition, and includes mechanistic considerations. No previous Review or Perspective exists on this topic. Previous literature was mainly limited to photodynamic thereapy (PDT) for cancer or inactivation of microbes with adjuvants such as natural products,^12^ chemotherapeutic agents,^13,14^ immunoadjuvants,^15^ metal–organic frameworks and dyes for thermal effects,^16,17^ plant extracts and essential oils,^18^ vitamin D and other differentiation-promotion compounds,^19,20^ and even hyperbaric oxygen.^21^ Reviews on PDT have also described iron-catalyzed reduction of lipid hydroperoxides and nitric oxide scavenging of peroxyl and alkoxy radicals.^22,23^ There are reviews on the use of PDT itself as an adjuvant in surgical resection^24^ and in fluorescence guided resection.^25^ Previous literature reviews have focused on photooxidation reactions, especially the organic chemistry of *singlet oxygen*.^26^ Thus, we believe that a Perspective is needed on photooxidation enhancement in the presence of additives/adjuvants with attention paid to the reactive species formed.

Most literature on bioactive agents is disconnected from the photosciences^27−34^*and* vice versa, literature on photooxidation is often disconnected from the influence of bioactive agents.^35−39^ There is less consideration of the two together, and also sparse consideration of RMS subsequent or prior to Type-I and Type-II photooxidation reactions. This Perspective is aimed to foster further research on the subject, and is largely a comparison of species that amplify RMS, which includes natural compounds and repurposed drugs in the coverage as mechanistic insights are sought.

## Scope

**Figure 3:**
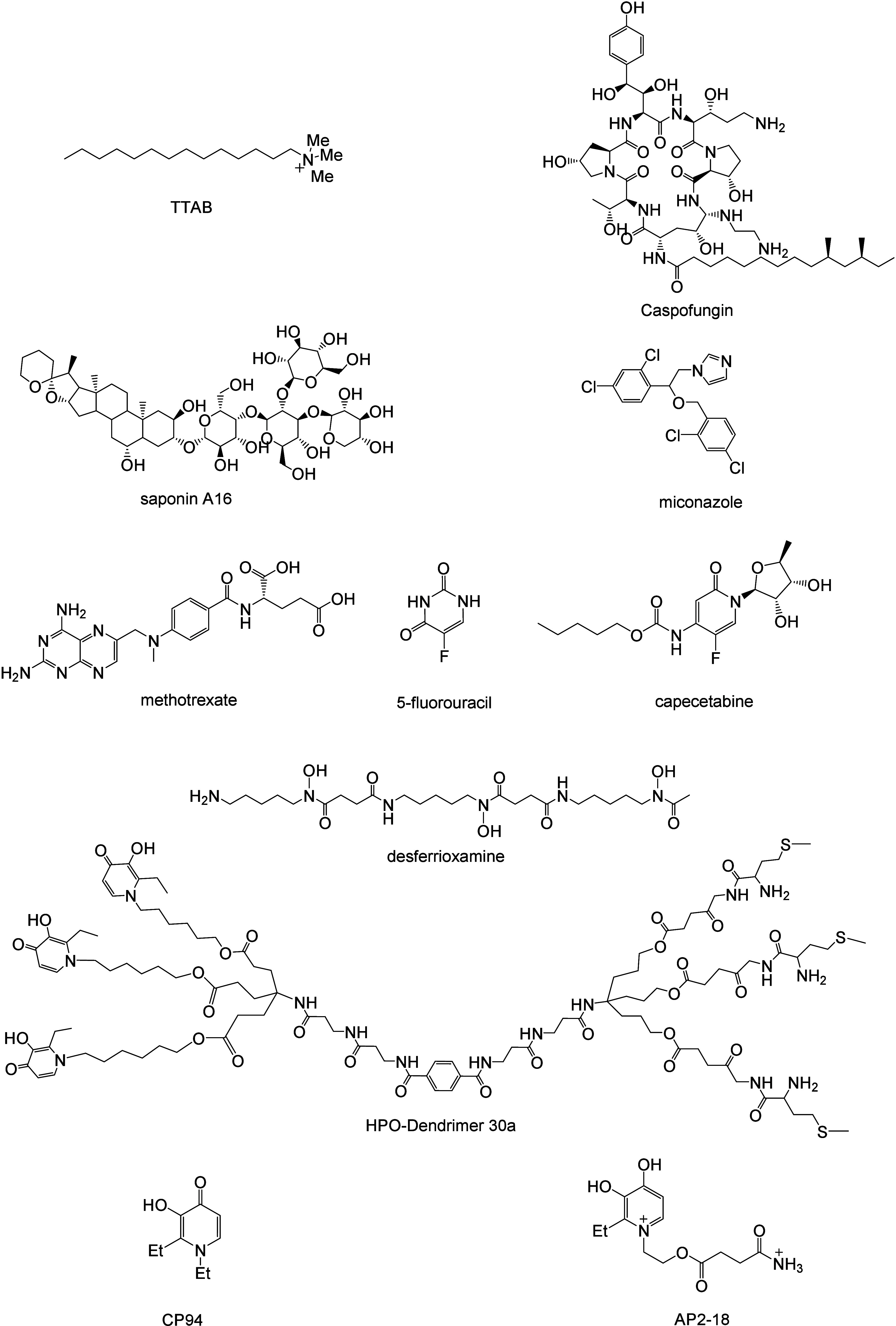
Structures of some additives discussed in this Perspective.

In this Perspective, we will discuss sensitized photooxidations in the presence of additives/adjuvants. The first section of the Perspective will include organic ^1^O_2_ reactions with additives that lead to oxidants that are more powerful than ^1^O_2_ itself. Subsequent sections of the Perspective will describe the reactive species generated, where data are available. The additives in these sensitized photooxidation reactions include organic sulfides, amino acids, lipids, natural products, repurposed drugs, and inorganic salts. Some of these additives are shown in Figure 3.

We will next compare sequential L-D and D-L pathways, where data are available. Different levels of confidence exist in understanding additive effects, where L-D vs D-L cycles have not yet been deconvoluted. Additives have displayed utility in significantly amplifying sensitized photooxidation reactions, giving us reason to embark on this Perspective.

Finally, we will discuss additive boosting of photooxidative activity as follows: in (1) chemical systems, erythrocyte membrane (ghost), and organic sulfide reactions, (2) cholesterol-enhanced PDT, (3) drug- and vitamin-enhanced PDT, (4) inorganic compound-enhanced photodynamic inactivation (PDI) of bacteria and fungi, and (5) drug- and surfactant-enhanced PDI.

### Amplified Reactivity from Post-irradiation Erythrocyte Lipid and Organic Sulfide ROS

**Figure 4:**
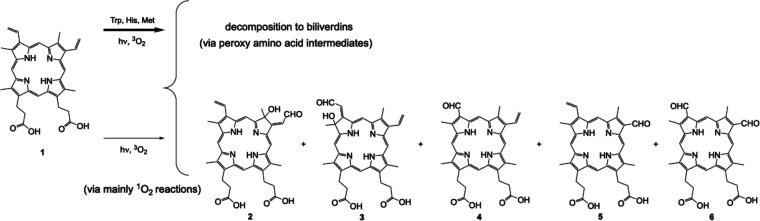
Products of protoporphyrin IX (PpIX) self-photooxidation.

**Figure 5:**
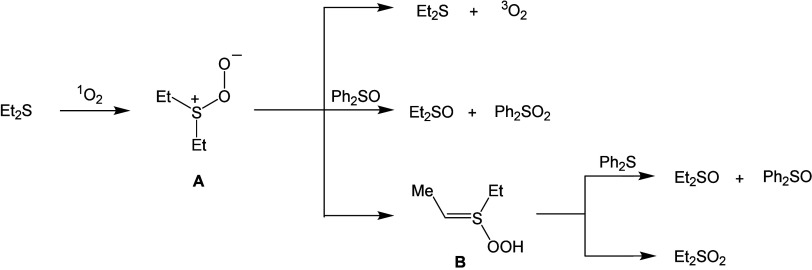
Reaction of diethyl sulfide with ^1^O_2_ in the presence of an additive (diphenyl sulfoxide or diphenyl sulfide). Under the reaction conditions, Ph_2_SO and Ph_2_S are themselves not reactive with ^1^O_2_.

What follows is a discussion of the boosting of reactivity with additives that arises in chemical systems after photooxidation reactions by an L-D reaction sequence (Figures 4 and 5, and Table 1). Here, we summarize instances of amplified photooxidative activity with amino acid additives (Trp, His, Met, Ph_2_S or Ph_2_SO).

**Table 1:** Additives That Amplify the Activity of ^1^O_2_

entry	system	additive and sensitizer	candidate RMS	enhancement	ref
1	microemulsion (oil-in-water)	Trp, His, Met, or erythrocyte “ghosts” (lipids) and PpIX	hydroperoxide, endoperoxide, persulfoxide	1.6-fold	(40)
				1.8-fold	
				2.0-fold	
				1.8-fold	
2	aprotic solvents	Ph_2_S or Ph_2_SO with alkyl sulfides and tetraphenylporphyrin (TPP)	Et_2_S^+^OO^–^, EtS(=CH_2_CH_3_)OOH	>10-fold	(44), (46)

In 1984, Krieg and Whitten^40^ reported that photooxidation of protoporphyrin (PpIX) was enhanced by the inclusion of certain amino acids and erythrocyte membrane (ghost) lipids (Table 1, entry 1). PpIX underwent self-sensitized photooxidation, producing hydroxyaldehydes (via [4 + 2] ^1^O_2_ cycloadditions), and formyl products (via [2 + 2] ^1^O_2_ cycloadditions) (lower arrow, Figure 4). The PpIX photooxidation was enhanced 1.6-, 1.8-, 2.0-, and 1.8-fold by the presence of tryptophan, histidine, methionine, and ghost lipids, respectively. These additives formed peroxy intermediates that converted the porphyrin into biliverdins by subsequent dark reactions. The enhancement was thought to arise from initial ^1^O_2_ reactions, but even more importantly from subsequent dark reactions from persulfoxides, endoperoxides, and hydroperoxides.^41,42^ These intermediate stabilities vary; for example evidence for the existence of persulfoxides comes from indirect trapping studies, whereas endoperoxides and hydroperoxides are often detected by NMR.

More recently, it was reported that reaction of organic sulfides with ^1^O_2_ can give rise to ROS that are strong oxidants (Table 1, entry 2).^43−51^ Alkyl sulfides [e.g., diethyl sulfide Et_2_S or thietane (CH_2_)_3_S] react with singlet oxygen to produce sulfoxides (Et_2_SO) and small amounts of sulfone (Et_2_SO_2_) (Figure 5). For the sulfide**–**^1^O_2_ reaction in the presence of diphenyl sulfoxide (Ph_2_SO) or diphenyl sulfide (Ph_2_S), Ph_2_SO_2_ or Ph_2_SO are formed, respectively. Because these aromatic compounds react very slowly with ^1^O_2_, the oxidation of Ph_2_SO and Ph_2_S was attributed to a peroxysulfoxide (**A**), a nucleophilic oxidant, and an *S*-hydroperoxysulfonium-ylide (**B**), an electrophilic oxidant), both shown to be stronger oxidants than ^1^O_2_ itself. In the following section, the use of a cholesterol-derived additive is discussed in the photooxidative eradication of cancer cells.

### Amplified Membrane Damage by PDT-Generated Lipid Hydroperoxides Such as Cholesterol Hydroperoxides (ChOOHs)

**Figure 6:**
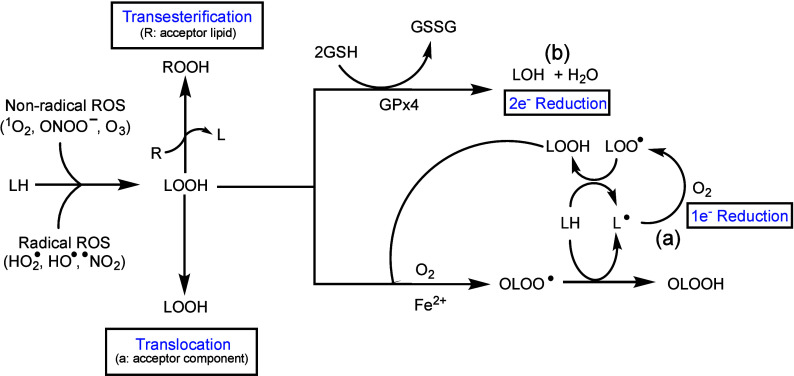
Formation and possible fates of lipid hydroperoxides (LOOHs). Selected free radical and nonradical initiators of lipid peroxidation are shown. The translocation of the LOOH to an acceptor mebrane or transesterification to an acceptor lipid is represented. Also shown is the turnover of lipid hydroperoxide (LOOH) intermediates via (a) one-electron reduction, which results in damage enhancement, and (b) two-electron reduction, which results in damage containment.

What follows is a discussion of lipid-specific L-D reaction sequence that can occur in cancer cells after a PDT challenge. Relatively low polarity photosensitizers such as PpIX can localize in lipoproteins or cellular membranes making them highly susceptible to photooxidative insults. Although damage to associated proteins can occur, unsaturated lipids such as phospholipids, glycolipids, and cholesterol are more prominent targets due to their overall prevalence. Lipid photooxidation or peroxidation (LPO) can be triggered by a free radical ROS such as hydroxyl radical (HO^•^) or by ^1^O_2_, the former generated by type I photodynamic reactions and the latter by type II reactions.^9,10^ Lipid hydroperoxide (LOOH) intermediates are generated in the process (Figure 6). A photogenerated radical can initiate LPO via allylic hydrogen abstraction from an unsaturated lipid, e.g. *sn-2* fatty acyl hydrogen in a phospholipid or carbon-7 hydrogen in cholesterol. The resulting lipid radical reacts rapidly with O_2_ to give a peroxyl radical (LOO^•^ in general, but 7-OO^•^ for cholesterol specifically). The LOO^•^ then triggers propagative LPO by abstracting hydrogen from another lipid, thereby becoming a hydroperoxide species [designated LOOH/ChOOH in general or 7α/β-OOH (7-OOH) as a specific positional ChOOH ].^9,52^ Photogenerated ^1^O_2_ adds directly to an unsaturated lipid to give LOOH with hydrogen retention and double bond allylic shift.^10^ For cholesterol, the major LOOH/ChOOH generated from ^1^O_2_ is 5α-OOH.^52^

In the presence of suitably ligated Fe^3+^ and a reductant such as superoxide or ascorbate, LOOHs can undergo 1-electron reduction to oxyl radical (LO^•^) intermediates which, either directly or after rearrangement with O_2_ addition to give epoxyallylic peroxyl radicals (OLOO^•^),^9,52^ abstract hydrogens from other lipids, thereby initiating chain LPO (Figure 6). This is an important example of a post-irradiation, light-independent process that can exacerbate the membrane-damaging effects of photodynamic action alone.^9,10,52^

Chain LPO induced by primary LOOHs can be suppressed by natural lipophilic antioxidants such as α-tocopherol and β-carotene, which intercept free radicals. However, primary and downstream LOOHs are usually susceptible to enzymatic 2-electron reduction, which prevents deleterious 1-electron turnover. The only enzyme known to catalyze redox inactivation of ChOOHs as well as PLOOHs in membrane environments is selenoperoxidase GPx4 (∼20 kDa), which uses glutathione (GSH) as a reducing cofactor.^53^ GPx4 is found in several compartments of mammalian cells, including cytosol and mitochondria, protecting against LPO-inflicted damage/dysfunction^54^ or a recently discovered form of cell death called ferroptosis.^54^

Damaging LPO due to 1-electron turnover of photogenerated cellular LOOHs is not necessarily restricted to their membranes of origin. It is now clear that ChOOHs, for example, can translocate to other membranes, and more rapidly than parent cholesterol due to greater polarity of the hydroperoxides.^55^ Intracellular translocation rate of ChOOHs is increased by trafficking proteins such as sterol carrier protein-2 (SCP-2) and proteins of the steroidogenic acute regulatory (StAR) family.^56,57^ SCP-2 is a nonspecific lipid transporter, whereas StAR proteins are specific for sterol-based lipids. Mammalian cells overexpressing SCP-2 internalized liposomal 7-OOH more rapidly than controls and died faster by apoptosis due to mitochondrial LPO and loss of membrane potential.^56^ More recent studies showed that StAR-mediated transport of 7-OOH along with cholesterol to mitochondria in steroidogenic cells caused damage/dysfunction that significantly reduced progesterone output.^58^ Chain peroxidation of mitochondrial lipids was again found to be mainly responsible. In the following section, the use of additives are discussed for amplified 5-aminolevulinic acid-PDT.

### Amplified 5-Aminolaevulinic Acid (ALA)-PDT in the Presence of Additives

**Figure 7:**
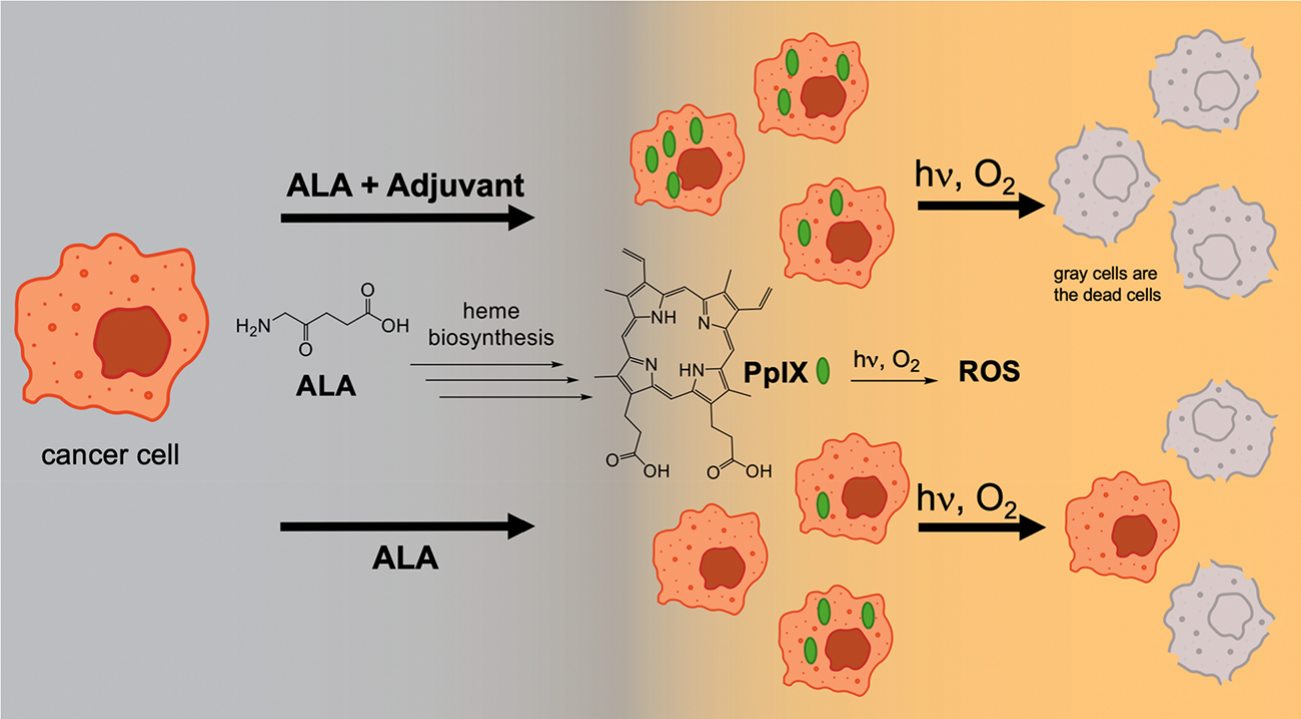
Topical administration of ALA that in the presence of an adjuvant (listed in Table 2) is followed by enhanced PpIX formation and greater photodynamic killing. This is a D-L reaction sequence.

This section summarizes instance of supportive roles of additives in ALA-photodynamic therapy mainly by an D-L reaction sequence (Figure 7 and Table 2). The adjuvants can lead to PpIX concentration increases and thereby enhanced photokilling of tumor cells. The adjuvants summaried are methotrexate, 5-fluorouracil, capecitabine, calcitriol, desferrioxamine, EDTA, 3-hydroxy-4-pyridinone (HPO) dendrimer, 1,2-diethyl-3-hydroxypyridin-4-one cation (CP94), as well as a conjugate of ALA and CP94 (ALA-hydroxypyridinone conjugate, AP2–18).

**Table 2:** Additive Boosting of Photodynamic Action by Increasing the PpIX Concentration in Cells

entry	cell type	adjuvant and pro-sensitizer	enhancement of the PpIX concentration	ref
1	prostate cancer LNCaP	methotrexate and ALA	3.0-fold (*in vivo*)	(59)
2	skin cancer A431 SCC13	methotrexate and ALA	3.0-fold (*in vivo*), 2.0–4.0-fold (*in vitro*)	(60)
3	skin cancer SCC A431	5-fluorouracil and ALA	3.5-fold (*in vivo*)	(61)
4	actinic keratoses	5-fluorouracil and ALA	2.0–3.0-fold (*in vivo*)	(62)
5	breast cancer BCA 4T1	capecitabine and ALA	4.0-fold (*in vivo*)	(63)
6	skin cancer SCC	calcitriol and ALA	2.0-fold (*in vivo*)	(64)
7	breast cancer MDA-MB-231	calcitriol and ALA	3.3-fold (*in vivo*)	(65)
8	skin precancer BCC	calcitriol and ALA	5.8-fold (*in vivo)*	(66)
9	larynx cancer Hep-2	desferrioxamine and ALA	1.9-fold (*in vitro*)	(67)
10	tongue cancer SCC15	desferrioxamine and ALA	1.2–1.5-fold (*in vitro*)	(68)
11	leukemic cells K562	EDTA and ALA	1.4-fold (*in vitro*)	(69)
12	breast cancer MCF-7R	HPO dendrimer and ALA	11-fold (*in vitro*)	(70)
	breast cancer MCF-7	HPO dendrimer and ALA	14-fold (*in vitro*)	(70)
	cervical cancer KB cells	HPO dendrimer and ALA	17-fold (*in vitro*)	(70)
13	skin cancer SCC A431	CP94 and ALA	2.1–4.4-fold (*in vitro)*	(71)
	fibroblasts 84BR	CP94 and ALA	1.5–5.0-fold (*in vitro*)	(71)
	skin cancer SCC A431	AP2-18	2.3–12.4-fold (*in vitro*)	(71)
14	glioma U-87 MG	CP94 and MAL	2.0-fold (*in vitro*)	(72)

Reports have appeared for methotrexate leading to enhancements of PpIX concentrations of 2–4 fold.^59,60^ 5-Fluorouracil also led to a 2.0–3.5 fold enhancement,^61,62^ while capecitabine led to a 4-fold enhancement,^63^ and calcitriol to a 2 5.8 fold enhancement.^64−66^ Reports have also appeared for desferrioxamine leading to enhancements of PpIX concentrations of 1.2–1.9 fold.^67,68^ EDTA also led to a 1.4-fold enhancement,^69^ 11–17 fold for the HPO dendrimer,^70^ 1.5–5.0 fold for CP94 as adjuvants in ALA-PDT.^71^ Also, a 2.3–12.4 fold enhancement of PpIX was shown for AP2–18.^72^ Lastly, a report appeared showing a 2.0 fold enhancement PpIX with CP94 as an adjuvant in methyl aminolevulinate (MAL)-PDT.^72^ The cell types studied in the *in vitro* and *in vivo* are also shown in Table 2.

The adjuvants in this section (Amplified 5-Aminolaevulinic Acid (ALA)-PDT in the Presence of Additives) led to 1.5–17 fold PpIX concentration increase in cells, in which corresponding cell photokilling increased from a low of 1.0 to a high of 49-fold. In a similar vein, Girotti et al., using ALA-PDT *in vitro*, have shown photokilling increases of 1.6 to 2.7-fold with adjuvants such as (i) *N*-(3-(aminoethyl)benzyl)acetamidine (1400W) and [2-[(1-iminoethyl)amino]ethyl]-*L*-homocysteine (GW271450), which are specific inhibitors of iNOS enzymatic activity, (ii) L-*N*^G^-nitroargininemethyl ester (L-NAME), a nonspecific NOS activity inhibitor, or (iii) 2–4-carboxyphenyl-4,4,5,5-tetramethylimidazoline-1-oxyl-3-oxide (cPTIO), a NO scavenger.^73,74^ The proposed mechanisms of adjuvant effects in ALA-PDT are often based not only on the effects of iNOS inhibitors or NO traps, but also on cell differentiation and proliferation, upregulation of coproporphyrinogen oxidase (CPO) and downregulation of ferrochelatase (FC), iron sequestration, and sometimes p53 protein expression to increase PpIX concentrations for enhanced cell photokilling. In the following section, the use of inorganic additives are discussed in regard to photooxidative eradication of microbes.

### Amplified Photodynamic Killing of Bacteria and Fungi in the Presence of Inorganic Additives

This section summarizes additives that enhance the photooxidized killing where L-D and/or D-L sequences may arise. The additives discussed are NaNO_2_, KSCN, KSeCN, and KI (Table 3).

**Table 3:** Additive Boosting of Photodynamic Inactivation of Bacteria and Fungi

entry	microbe	additive and system	candidate RMS and other species	photokilling enhancement	ref
1	*E. coli*, *S. aureus*	NaNO_2_ (100 mM) and RB (0.01 mM)	type I and type II ROS, NO_2_^•^, and O_2_NOO^–^	>6 log, 2–3 log	(75)
2	*E. coli*, *S. aureus*	KSCN (10 mM) and MB (0.01 mM)	mainly type II (^1^O_2_) and SO_3_^•–^	3.5 log, 2.5 log	(76)
3	*E. coli*, *S. aureus*	KSeCN (50 mM) and MB (0.01 mM), RB (0.2 mM), TPPS_4_ (0.2 mM), or SeCN (1, 10, or 50 mM) and MB (0.001 mM), RB (0.01 mM), or TPPS_4_ (0.0002 mM)	type I and type II ROS, (SeCN)_2_, and (SeCN)_3_^–^	>6 log, 3.0–5.0 log	(77)
4	*E. faecalis*	KI (50 mM/100 mM) and MB (4 × 10^–4^ M)	mainly type II (^1^O_2_), HOO^•^, H_2_O_2_, I_2_^•–^, and I_3_^–^	2 log (at 50 mM KI), 7 log (at 100 mM KI)	(78)

In 2019, it was reported that the rose bengal (RB)-sensitized photoinactivation of *E. coli* and *S. aureus* was substantially enhanced by NaNO_2_ (Table 3, entry 1).^75^ The photokilling enhancement was up to 6 log for *E. coli* and 2–3 log for *S. aureus*. The proposed mechanism involved Type-I and Type-II ROS (eq 1–3). Furthermore, ^3^RB is thought to react with nitrite (NO_2_^–^) to produce nitrogen dioxide radical (NO_2_^•^) and RB radical anion (RB^•–^), reaction of the latter with O_2_ giving superoxide anion (O_2_^•–^). It was also proposed that the peroxynitrate (O_2_NOO^–^) is produced and participates in the toxicity.
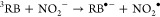
1
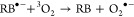
2
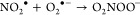
3

In 2013, St. Denis et al.^76^ described the photodynamic inactivation of *E. coli* and *S. aureus* in the presence of KSCN (Table 3, entry 2). The enhancement in photokilling by KSCN was 2.5 log for in *S*. *aureus* and 3.5 log for in *E. coli*. The mechanism for heightened activity was proposed to be a combined effect from ^1^O_2_ and SO_3_^•–^, the latter being generated by the singlet oxygenation of SCN^–^ and further oxidation of HSO_3_^–^ anion (eq 4–8).
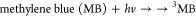
4
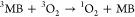
5
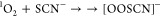
6
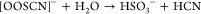
7
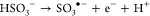
8

A subsequent report described the RB-, MB-, and 5,10,15,20-tetrakis(4-sulfonatophenyl)porphyrin tetraanion (TPPS_4_)-sensitized photoinactivation of *E. coli* and *S. aureus* in the presence of KSeCN (Table 3, entry 3).^77^ In the presence of KSeCN, up to a 6 log enhancement of photokilling was observed for *E. coli* and a 3–5 log enhancement for *S. aureus*. The proposed mechanism involved Type-I and Type-II ROS and products resulting from KSeCN oxidation, including the pseudohalogen selenocyanogen [(SeCN)_2_] and triselenocyanate anion [(SeCN)_3_^–^], which are thought to be antibacterial agents (eq 9 and 10).
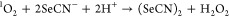
9
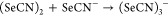
10

More recently, Yuan et al. described the MB-sensitized oxidation of *E. faecalis* in the presence of KI (Table 3, entry 4).^78^ The enhancement in photokilling was 2–7 log for *E*. *faecalis*. The combination of MB and KI was found to possess killing effects in an L-D sequence, where postillumination incubation was carried out under hypoxic conditions. The proposed mechanism involves the production of I_3_^–^ from the reaction with ^1^O_2_ with I^–^; the total quenching rate constant (*k*_T_) of ^1^O_2_ for I^–^ is 9.1 *x* 10^7^ M^–1^ s^–1^.^79^ Other participating active agents likely included I_3_^–^ and H_2_O_2_, and possibly I_2_^•–^ and HOO^•^ (eq 11–15). Continuing interest in the use of iodide in photodynamic inactivation of bacteria has been reported.^80^ In the following section, more examples of additives are discussed in photooxidative eradication of microbes.
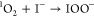
11
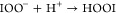
12
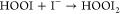
13
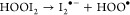
14
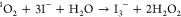
15

### Amplified Photodynamic Killing of Bacteria and Fungi in the Presence of Drugs or Surfactant Additives

This section summarizes additives that enhance the sensitized log killing by L-D and/or D-L sequences, such as integrity of the cell membrane compromised for greater cell permeability. The additives discussed are OPE–TTAB complex (oligo-*p*-phenylene ethynylene tetradecyltrimethylammonium cation), saponin A16, caspofungin, miconazole, and 8-methylnon-7-ene-1 sulfonate (Table 4).

**Table 4:** Additive Boosting of Photodynamic Inactivation of Bacteria and Fungi

entry	microbe	additive and system	candidate RMS and other species	enhancement	ref
1	*E. coli*, *S. aureus*	OPE (0.01 mM) and TTAB (0.04 mM)	mainly type II (^1^O_2_)	3 log, 2 log	(81)
2	*C. neoformans*	caspofungin (8 μg/mL) and PEI-chlorin e6 (0.01 mM)	type I and type II ROS, cell wall integrity reduced, sensitizer localization increased	2.0 log	(82)
3	*C. albicans*	saponin A16 (4 μg/mL) and RB (0.1 mM), chlorin e6 (0.1 mM), or PEI-chlorin e6 (0.01 mM)	type I and type II ROS, cell membrane integrity reduced, sensitizer localization increased	2.0–5.0 log for RB or chlorin e6	(83)
4	*C. albicans*	miconazole (25 μg/mL) and TMPyP (10 μg/mL)	type I and type II ROS, and additional enzymatic oxidative stress	0.3 log	(84)
5	*E. coli*	8-methylnon-7-ene-1 sulfonate (1.0 mM) and airborne ^1^O_2_	airborne ^1^O_2_, “ene” hydroperoxides, and their decomposition products	0.23–0.30 log	(85), (86)

About ten years ago, it was reported that oligo-*p*-phenylene ethynylene (OPE)-sensitized photodynamic inactivation of *E. coli* and *S. aureus* could be significantly enhanced by tetradecyltrimethylammonium bromide (TTAB) as an OPE–TTAB complex (Table 4, entry 1, eq 16–18).^81^ The enhancement in photokilling was 3 log in *E. coli* and 2 log in *S. aureus*. The proposed mechanism involved a combination of the membrane-disrupting effect of anionic OPE and the photogenerated ROS. The formation of the positively charged OPE-TTAB complex allows anionic OPE to enter the bacterial cell membrane. The OPE-TTAB complex dissociates in the membrane due to the stronger interaction of TTAB with lipids. The now exposed anionic OPE experiences strong electrostatic repulsion with the negatively charged lipid bilayer resulting to membrane disruption and leakage. The repulsion may also cause the OPE to have greater access to the bacterial cytoplasm or periplasm, where it can cause further damage through ROS photogeneration.
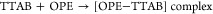
16
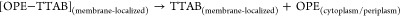
17
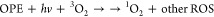
18

An earlier study described the polyethylenimine (PEI)-chlorin-e6-sensitized photinactivation of *Cyrptococcus neoformans* in the presence of caspofungin (Table 4, entry 2).^82^ A 4.0 log enhancement of cell kill was observed with caspofungin. The mechanism was thought to be due to Type-I and Type-II ROS along with caspofungin’s inhibitory effect on (1,3)β-d-glucan synthase, leading to the reduction of (1,3)β-d-glucan, thus compromising cell wall integrity, leading to a greater uptake of PEI-chlorin e6.

In 2010, a report appeared on the RB, chlorin-e6, and polycationic polyethylenimine/chlorin e6 conjugate (PEI-chlorin-e6)-mediated photodynamic inactivation of *Candida albicans* in the presence of the natural product, saponin A16 (Table 4, entry 3).^83^ The enhancement in the photokilling was 2.0–5.0 log with RB and chlorin-e6 with saponin A16. The proposed mechanism involved greater production of Type-I and Type-II ROS due to cell membrane disruption by saponin A16, thereby increasing RB and chlorin-e6 uptake. The polycationic charge of PEI-chlorin e6 facilitated its entry into the fungal cell; thus no enhancement in photokilling was observed in the presence of saponin A16.^83^

A report in 2010 described 5,10,15,20-tetrakis(1-methyl-4-pyridinio)porphyrin tetracation (TMPyP)-sensitized photodynamic inactivation of fungus *C. albicans* in the presence of miconazole. Inclusion of the latter led to a 0.3 log enhancement in fungal killing (Table 4, entry 4).^84^ Mechanistically, it was proposed that miconazole’s effect was due to its ability to inhibit mitochondrial ATPase and mitochondrial respiration, resulting in the formation of additional ROS and thereby enhancing the oxidative stress of PDT.

**Figure 8:**
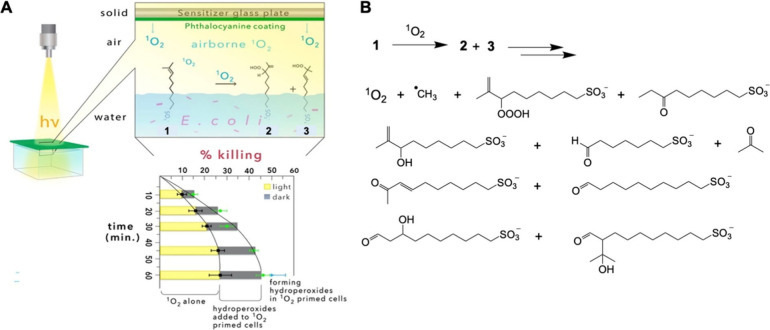
Percent of *E. coli* killed by airborne ^1^O_2_ alone (yellow bars) and the additional percent of *E. coli* killed upon adding hydroperoxides **2** and **3** in the dark as a follow-up treatment (gray bars). This is an L-D reaction sequence.

More recently, there were two reports dealing with airborne ^1^O_2_-mediated inactivation of *E. coli* in the presence of alkene surfactant **1** by an L-D reaction sequence (Figure 8, Table 4, entry 5).^85,86^ The enhancement in photokilling was 0.23–0.30 log for *E. coli*. The proposed mechanism was based on the toxicity of airborne ^1^O_2_ itself (*k*_T_ for ^1^O_2_ with alkene surfactant **1** at the air–water interface is 1.1 × 10^6^ M^–1^ s^–1^), and in the dark subsequent toxicity from ROS due to allylic hydroperoxide decomposition likely forming hydrotrioxide, methyl radical, additional ^1^O_2_. This is thought to be a ^1^O_2_ priming reaction and subsequent contribution from ROS in the dark due to the oxidized additive decomposition. Interestingly, hydroperoxide products **2** and **3** are of very low toxicity on their own. Rather, they become toxic only *after* the bacteria are pretreated with ^1^O_2_ singlet oxygen. Thus, there is a “one-two” punch, where the alkene surfactant has a strong secondary toxic effect, due to its light-independent (dark) reaction.

## Mechanisms

**Figure 9:**
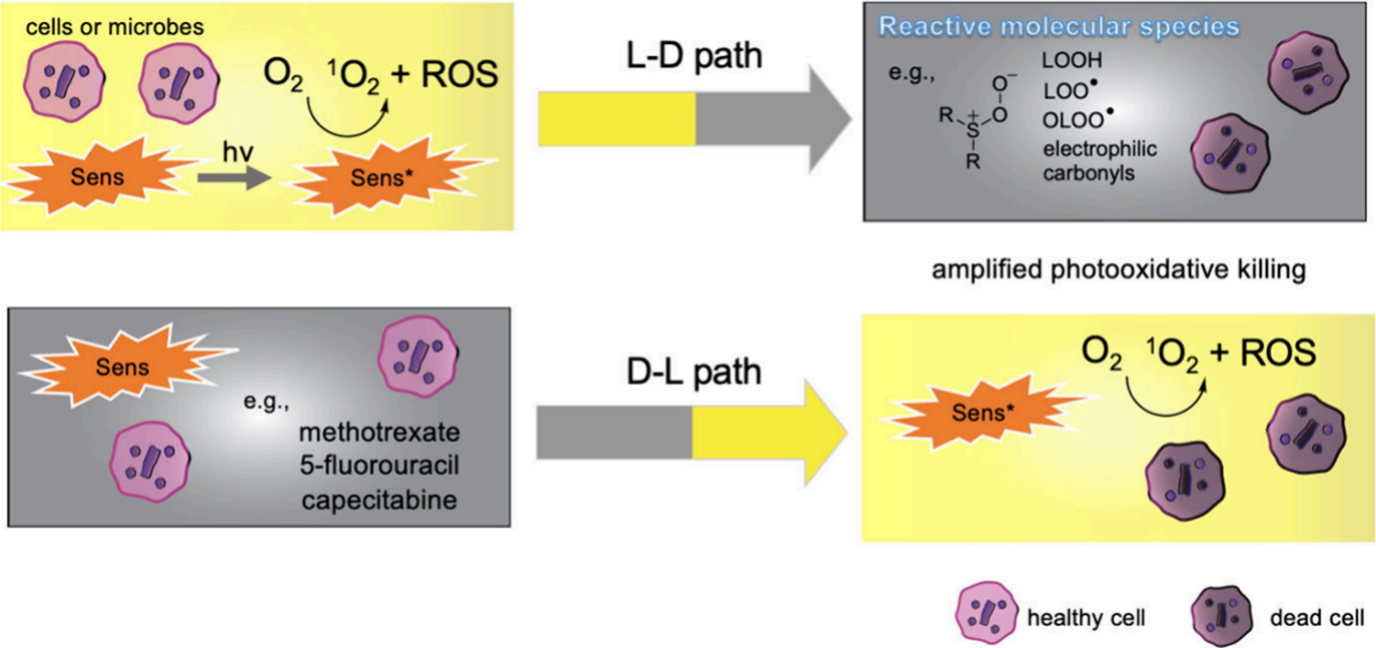
Mechanistic summary of L-D and D-L reaction sequences leading to overall greater photooxidative damage.

After photooxidation forming HO^•^, O_2_^•**–**^, HO_2_^•^, and H_2_O_2_ in Type-I reactions, and ^1^O_2_ in the Type-II reaction, the presence of additives can lead to subsequent RMS. The formation of RMS arises not only in L-D but also D-L sequences causing greater photooxidative damage (Figure 9). It is valuable to highlight how this L-D and D-L reaction sequence with additives boosts photooxidative activity. We highlight examples in which (1) amino acids and lipids enhance the net oxidizing power in photooxidation reactions, mainly attributed to peroxy intermediates formed downstream some with greater destructive power than ^1^O_2_ itself. Such post-irradiation organic sulfide ROS included persulfoxide R_2_S^+^OO^–^ and hydroperoxy-sulfonium ylide RS(**=**CH_2_R)OOH. (2) Cholesterol-enhanced PDT arises by post-irradiation membrane damage/dysfunction caused by PDT-generated lipid hydroperoxides. While primary membrane LOOHs may be deleterious on their own (depending on levels attained), they could induce secondary or after-light LPO which is potentially more damaging and cytotoxic. This may be due to light-independent redox turnover of primary photogenerated LOOHs.^87^ This turnover, which results in damaging LPO, could occur in originating membranes or in others to which primary LOOHs may have been transferred. (3) For ALA-PDT, a variety of adjuvants have shown success, including repurposed drugs, vitamins, and iron chelating agents. This is clearly due perturbation of heme biosynthesis by a D-L path increasing CPO and decreasing FC, leading to increased amounts of PpIX. Enhancement in PpIX concentrations were up to 17-fold *in vitro* and 5.8-fold *in vivo*. (4) Table 3 shows that inorganic compounds lead to amplified photodynamic of up to 6 or 7 log killing of bacteria or fungi. This amplification of the photosensitized activity is attributed to RMS including NO_2_^•^, O_2_NOO^–^, [OOSCN]^−^, SO_3_^•–^, [(SeCN)_2_], [(SeCN)_3_^–^], I^•^, I_2_^•–^, I_3_^–^, and HOOI. These RMS will of course have a varying range of lifetimes and reactivities. Thus, compounds and cell constituents otherwise unreactive to ^1^O_2_ may be decomposed. (5) Drugs can react by destabilizing cell walls and membranes (caspofungin and saponin A16), inhibiting DNA synthesis (5-fluorouracil), or sequestering iron (desferrioxamine and HPO dendrimer) to amplify damage pre- or postphotodynamically. Amplified photodynamic killing enhanced PDI is found with surfactant additives, where dark toxicity from ROS due to allylic hydroperoxide decomposition likely forming hydrotrioxide, methyl radical, additional ^1^O_2_ based on the proposed mechanism in Figure 8. The state-of-science is currently limited to classes of additives that have been covered here. Future screening of additives that are, e.g., susceptible to peroxidation, could allow for a rational selection with downstream RMS mechanistic pathways in mind. Broader classes of additives could be tested for deeper insight into predicting well-performing additives.

## Conclusions

An importance of this Perspective is emphasized, namely that not only for organic chemists, but also photobiologists, that there is a need to know more about underlying mechanisms to assist in therapies like PDT. We now report on progress in understanding of mechanistic details of additives to amplify photooxidation reactions. Greater oxidizing power is sometimes caused by the additives. The generation of RMS to enhance photooxidation was discussed, where the RMS are often tentatively assigned. A challenge is the direct detection of RMS arising from additives, which form in downstream reactions.

Challenging problems that await exploration include: (i) the design of photoreactions to deconvolute the light and dark paths, to have better amplification control, (ii) further development of techniques to monitor RMS in cells and microbes, (iii) examination of wider range of additives, including sensitizers as oxidizable substrates themselves, (iv) to assess the amplification of photooxidation based on RMS vs drugs to provoke different mechanisms, (v) establishing additive roles both as adjuvant and converse, e.g., Fe chelators where low amount (promotes radical generation and oxidative stress via Fenton-like reactions), and high amount (starves the organism of this essential metal making it weaker and more susceptible to photodynamic stress). Efforts are needed to capitalize further on additives for an understanding to amplify photooxidative reactions. Thus, we believe that exploring additive-based photodynamics and mechanisms of action has a bright future in light as well as dark reactions.

## Data Availability

The data underlying this study are available in the published article.
